# Synergy of Arsenic and Graphene Oxide in Utero and Lactation Exacerbates Reproductive Disorders in Female Rat Offspring Undergoing Puberty and Maturity

**DOI:** 10.3390/toxics13090787

**Published:** 2025-09-17

**Authors:** Reda H. ElMazoudy, Azza A. Attia, Tawfik A. Saleh

**Affiliations:** 1 Zoology Department, Faculty of Science, Alexandria University, Moharram Bek, Alexandria 21511, Egypt; 2 Department of Chemistry, King Fahd University of Petroleum and Minerals, Dhahran 31261, Saudi Arabia

**Keywords:** sodium arsenite, nano-graphene oxide, puberty, anogenital distance, vaginal opening, estrous cycle, sexual behaviour

## Abstract

Notably, the widespread ubiquity of arsenic and graphene oxide in the environment validates the occurrence of their co-exposure, posing significant threats to target organisms, including humans. Herein, prepuberty, puberty, and maturity were investigated using anogenital distance, vaginal opening, first estrus, reproductive hormone profiles, cyclicity, sexual behaviour and pregnancy outcomes to assess the impact of exposure to arsenic and/or graphene oxide on the puberty of offspring female rats after maternal exposure during gestation and lactation periods. Zero-day pregnant Sprague Dawley females were randomly divided into four groups, each receiving a different treatment via drinking water from gestation day 0 to postnatal day 21: control group (CON, drinking water); arsenic group (ARS, 10 mg/L sodium arsenite); graphene oxide group (GOX, 0.5 mg/mL); and co-exposure group (ARS + GOX; 10 mg/L of arsenic combined with 0.5 mg/mL of graphene oxide). Individually or combined, arsenic and graphene oxide exposure increase the sexual retardation and female masculinization, as evidenced by a significant increase in anogenital distance, delay in the first estrus cycle, and prolongation in the timing of the vaginal opening. At maturity, the offspring exhibited a significant elevation of testosterone and a significant decrease in estradiol. Offspring females showed inhibited receptivity to their male mates, indicated by lower lordosis quotient and intensity. Additionally, there was an increase in the number of estrous cycles but a decrease in their duration. Moreover, an increase in implantation loss and the number of resorbed embryos, along with a reduction in viable fetuses. In conclusion, reproductive deterioration was more significant in the offspring exposed to combined arsenic and graphene oxide compared to those exposed to ARS or GOX alone, indicating that arsenic exposure is exacerbated when combined with graphene oxide during the experimental episode.

## 1. Introduction

Co-exposure to various environmental pollutants, whether natural or anthropogenic, is an increasing concern that poses significant health risks to humans, which are significantly exacerbated by the prevalence of nanomaterials [1]. Both humans and animals are the primary victims of environmental toxicants, often suffering from excessive exposure. On the other hand, the developing reproductive system is susceptible to perturbations induced by excessive co-exposure to environmental toxic factors, leading to reproductive injury in puberty and fertility [2].

Arsenic (ARS), a heavy metal, is one of the most ubiquitous metalloids and environmental toxicants, with a pervasive existence in drinking water, foods, and soil from various sources. Smelting, refining, and mining activities are also common anthropogenic sources of arsenic deposition, increasing environmental contamination [3]. Additionally, occupational exposure and extensive intake of arsenic can result from excessive application in agriculture and the manufacturing of pharmaceuticals, glass, wood, and food preservatives [4].

Arsenic, despite its well-known toxic effects in humans, has been extensively documented in the scientific literature as affecting or contributing to alterations in developmental parameters, particularly through its elevated concentrations in human blood serum. Moreover, toxic exposure to arsenic has increased in recent years, partly due to the intensified environmental and dietary exposure to this metalloid [5].

The capability of arsenic to cause toxicity is well known in acute and chronic exposure, causing numerous disruptions. Indeed, research has clearly documented that arsenic is a potent endocrine disruptor with a high affinity to cross and accumulate in tissue barriers, such as hypophysis–pituitary–gonads, blood–gonads, blood–placenta, and blood–breast [6,7,8]. Cumulative animal studies indicate that arsenic causes gonadotoxicity upon early-life exposure (prenatal, postnatal, and extending to the pubertal stage) [9,10].

Confirming this, it has been shown that arsenic exposure disturbs levels of reproductive hormones, leading to an irregular estrous cycle in female rats [11,12]; delays the onset of vaginal opening [13]; and disrupts the normal development of the mammary gland in the pubertal stage. In addition, a reduction in the number of nipples/areolas and a decrease in the anogenital distance are the most characteristic symptoms in female offspring exposed to arsenic in utero [14].

Extensive use in modern applications, such as bioremediation, biomedicine, electronics, and the agricultural industry, has triggered the significant release of graphene oxide (GOX) into the environment. Toxicologically, this emission of graphene oxide may pose a threat to human health and the ecosystem [15]. The interaction of graphene oxide with organic and inorganic substances is key to the risk of coexposure. Hence, in most cases, the coexposure can result in synergistic/antagonistic effects [16].

Few studies have investigated graphene oxide (GOX) nanotoxicity data; these have mainly been conducted on mammals, amphibians, zebrafish, and fungi. An accumulating number of reports have demonstrated that graphene oxide can interact with biological macromolecules, such as nucleotides, proteins, and polysaccharides, resulting in substantial risks in tissues and organs [17]. Other reports have shown that graphene oxide exposure results in the impairment of embryogenesis of zebrafish [18], inducing oxidative stress [19] and genotoxicity [20]. Malformations, mortality, and birth defects were also evident in embryos and juveniles following prenatal exposure to graphene oxide [21].

Aqueous aggregation processes can significantly impact function, effective toxicity, environmental transport, and fate of advanced nanoscale materials, including graphene and graphene oxide (GOX) [22].

The present study focuses on the impacts of the significant prevalence of arsenic in drinking water and the occupational exposure to graphene oxide on the reproductive system. It aims to assess the potential effects of arsenic coexisting with graphene oxide on the developmental puberty in female offspring through a simulated experimental approach to environmental exposure.

This study hypothesizes that the findings will provide valuable insights into the combined effects of the nano-graphene oxide and arsenic toxicity on the prepuberty, puberty, and maturity of female offspring during gestation and lactation.

## 2. Materials and Methods

### 2.1. Chemicals

#### 2.1.1. Sodium Arsenite

Sodium arsenite (NaAsO_2_, CAS No. 7784-46-5, CAT No. 71287, EC No. 232-070-5) was purchased from Sigma-Aldrich (St. Louis, MO, USA) and dissolved in drinking water (vehicle).

#### 2.1.2. Graphene Oxide

##### Synthesis of Graphene Oxide

Graphene nanosheets were synthesized using the method of Hummers and Offeman [23]. Around 6 g of graphite was mixed with 3.6 g of potassium peroxodisulfate and 3.6 g of phosphorus pentoxide. After homogenous mixing, the obtained mixture was added to 150 mL of sulfuric acid in an ice bath under continuous stirring. After 3 h, the system was heated and refluxed at 80 °C in the oil bath for 24 h. The system was then allowed to cool, and the obtained material was filtered by centrifuge, washed, and dried. Then, 90 mL of H_2_SO_4_ was added to the solid material by stirring in an ice bath. 16 g of potassium permanganate was added while stirring. After 2 h, 0.180 mL of distilled water was added dropwise. After a while, 30% H_2_O_2_ was added to the solution until a yellowish colour appeared. The mixture was kept under stirring for 12 h. The graphene was then filtered by centrifuging. Then it was washed with diluted HCl (1/10 *v*/*v*). The graphene was then dried. The solution of graphene oxide was then prepared as required for testing.

##### Characterization Analysis of Graphene Oxide

Fourier transform infrared spectrum with a wavelength range 500–4000 cm^−1^ was run on a Thermo Scientific Nicolet 6700 FTIR spectrometer (Waltham, MA, USA) to investigate the graphene structure. Sample pellets were prepared by mixing 1% graphene with KBr using the Atlas™ (Atlas Autotouch/Atlas™, Specac Ltd./Specac Inc., Washington, PE, USA) and then transferred into an FTIR cell for analysis. The surface morphology of the sulphide material was recorded on a Field Emission Scanning Electron Microscope FESEM (TESCAN, LYRA 3, FESEM/FIB-SEM, Brno, Czech Republic) at an accelerating voltage of 20 kV. A transmission electron microscopy (TEM) image was obtained using a JEM-2100F Field Emission Electron Microscope (JEOL-USA, Inc., Peabody, MA, USA) at 200 kV acceleration voltage. HORIBA Scientific Lab RAM HR (HORIBA France SAS, Palaiseau, France) Evolution Raman spectrometer was used to accumulate the Raman spectrum of graphene. The UV-visible spectra were obtained by a Genesis 10S UV-Vis pectrophotometer (Thermo Scientific (Thermo Fisher Scientific Inc., Waltham, MA, USA), using a standard quartz cuvette at room temperature, 200–700 nm.

### 2.2. Sodium Arsenite Dose

The arsenic dose (10 mg/L) is based on the study of developmental and reproductive toxicity in previous studies of Aquino et al. [24] and data from the World Health Organization (WHO) [25]. This dose is equivalent to the normalized human dose of 1.6 mg/L by body surface area [26] for the arsenic-exposed regions [27].

### 2.3. Graphene Oxide Dose

0.5 mg/mL obtained from (500 mg/L) stock drinking water-suspended solution of nano-graphene oxide (GOX). The concentration of graphene oxide is based on studies of developmental and reproductive toxicity [28]. Drinking water was administered in the negative control group in the same pattern as the treated groups. Both doses of sodium arsenite and graphene oxide were bi-weekly refreshed.

### 2.4. Ethics Statement

All the experimental protocols of Animal Use, Care and Welfare were certified and supervised by the Institutional Animal Use and Care Committee of the Faculty of Science (IACUC), Alexandria University (Serial Number: AU04/605/a26-8-25). The experimental procedures strictly adhered to the Good Laboratory Practice Guidelines and the applicable animal welfare legislation set forth by the OECD [29], as well as the guidelines provided by the NRC [30] concerning environmental stress and distress in laboratory rats. All animals were treated humanely, with a focus on alleviating their suffering.

### 2.5. Animals Fetch

Sixty 90-day-old (150–170 g weight) adult nulliparous female rats and thirty 80-day-old (140–160 g weight) proven-fertile male rats of the Sprague Dawley strain from a breeding stock colony were provided by the Centre of Animal Husbandry (Rearing Animal Centre) of the Faculty of Agriculture, Alexandria University, Chatby, Alexandria, Egypt. The animals are housed in polypropylene cages with absorb-dry and care fresh bedding and maintained under-ventilated controlled conditions (24 ± 2 °C, 60 ± 10% humidity, and a cycle of light/dark (on/off 07.00) with free access to chow food and water ad libitum. There was no evidence of disease after gross inspection of both female and male rats. Female and male rats were acclimated and quarantined for two weeks.

### 2.6. Mating and Pregnancy Layout

For preparation of the mating experiment, nulliparous female rats cohabited with males (2 females/1 male/cage) and received free food and drinking water ad libitum. Females were timed at zero days of gestation (GD0) on detection of sperm plugs in the vaginal cervix. Following the mating, the positively inseminated females were individually housed in separate cages for the treatment protocol [31].

### 2.7. Experimental Assignment and Design

Zero-day pregnant Sprague Dawley females were indiscriminately allocated into four experimental groups (*n* = 15 females/group): control group (CON, drinking water), arsenic group (ARS, 10 mg/L sodium arsenite), graphene oxide group (GOX, 0.5 mg/mL), and co-exposure of graphene oxide and sodium arsenite group (ARS, 10 mg/L + GOX, 0.5 mg/mL). The drinking water, sodium arsenite, and graphene oxide were administered daily ad libitum to pregnant female rats.

Pregnant females were individually housed in cages; the exposure occurred between GD1 and GD20 and was maintained throughout the lactation period (postnatal day 21, PND 21). The intrauterine period (GD1 to GD20) corresponds to the development of the hypothalamus and sexual differentiation in rats. Only neonatal female pups from each litter were kept for lactation until weaning on postnatal day 21. Following weaning, two weaned female offspring per littermate were randomly assigned and separately housed in individual cages (*n* = 2 female pups/cage) to avoid littermate intervention [32]. Thereafter, female offspring were maintained through puberty and maturity for reproductive evaluation (PND 30, 65, 90). The offspring subgroups comprised 120 female pups (30 female pups/each). Five separate replicates of each group were recorded.

### 2.8. Experimental Milestones

#### Maternal Toxicity Monitoring

The start and final maternal body weights, neurotoxicity, behaviour, and physical toxicity (hair loss, bleeding, aggression, and diarrhea) were registered as significant symptoms of maternal toxicity.

### 2.9. Female Offspring

#### 2.9.1. Measurement of Body Weight and Anogenital Distance

On postnatal day 1 (PND 1), female offspring from each group were weighed. Measurements of anogenital distance (AGD) were taken using a digital vernier calliper by applying the procedures described by Gallavan et al. [33]. The AGD is the distance from the centre of the anus to the posterior edge of the genital papilla. Anogenital index (AGI) was estimated by normalization of AGD to the cubic root of body weight.

#### 2.9.2. Determination of Location and Count of Nipples and Areolae

On PND 15, the mean number of nipple buds (teats) of female offspring (*n* = 30 female pups/group) was estimated in the epidermal region of the nipples by observation of nipple buds. Also, the number of areolae was counted by observation of a particular ring of pigmented skin surrounding a nipple or a skin discolouration [34].

#### 2.9.3. Estimation of Onset of Puberty

##### Vaginal Opening, First Estrus, and Ovulation

From pre-puberty days (PND 21–29), weaned female offspring from control and experimental groups were checked daily at 7:00 a.m. for the start of vaginal opening (VO). Under macroscopical examination, VO is fully gaping, and the tissues are swollen, moist, and reddish pink with visible longitudinal indentations in the dorsal and ventral lips of the vulva [35]. In puberty, samples of vaginal cytology swab smear were collected at 7:00 a.m. and spread thinly on a microscope slide for cytological examination to investigate the onset of the first proestrus or estrus stage (cornified epithelial cells). Examining vaginal smears continued until the detection of many neutrophils in the smear, with the first diestrus phase being detected, indicating the first ovulation [36].

#### 2.9.4. Scrutiny of Reproductive Cycle (Estrous Cycle) (Maturation Phase)

Starting from the first ovulation and for 15 days (PND 75–90), the four phases of the estrous cycle were estimated via a collection of vaginal smears. Briefly, each phase of the estrous cycle (proestrus/estrus and metestrus/diestrus) was indexed by the basic criteria (proportion, density, and arrangement) of three exfoliated cell types in vaginal smears (keratinized cells, leukocytes, neutrophils, and nucleated epithelial cells). Thereafter, the status of the cycling index (length, frequency, contiguity) and the duration of each phase were determined [37].

#### 2.9.5. Assessment of Lordosis and Sexual Receptivity

At sexual maturity (PND 90), only cycling females from each group were acclimated and habituated for one estrous cycle on an inverted light cycle (12 h light/dark) with food and water ad libitum for 5 days preceding the sexual behaviour test. Copulatory behaviour (lordosis) and receptivity were assessed following four hours of proestrous rats. Females are individually paired with vigorous stud and sexually adapted male rats in a Plexiglas observation cage during the day dark phase (10.00–12.00 p.m.) [38]. Ten mounts from experienced males were recorded. The lordosis quotient was calculated, as well as lordosis intensity, in four scales.

#### 2.9.6. Test of Natural Mating for Fertility and Reproductive Performance

All fertility tests and reproductive performances complied with the protocol of Housing, Caring and Mating Laboratory Animals [39]. After four hours of the sexual behaviour test, the individually coupled estrous female (PND 90) (*n* = 20/group) and male in the same cage were maintained overnight to assess the different parameters of the fertility test (natural mating). On GD20, pregnant females were euthanized and dissected. Each gravid uterine horn was opened, and the fetuses were further assessed and evaluated. The number of implants, corpora lutea, and fetal index (alive, dead, resorbed) were determined.

#### 2.9.7. Assessment of Body, Ovary, and Uterus Weights at PND 90

The remaining female offspring (*n* = 10/group) were weighed and killed by decapitation at the estrus phase. Thereafter, both ovaries and uteri were dissected out, cleared of attached adherent tissues (adipose and fascia), washed in PBS (phosphate-buffered saline), and weighed (wet organ weight).

#### 2.9.8. Reproductive Hormone Assays

For hormonal analysis, the blood samples were collected from the dorsal aorta in the same estrus phase and centrifuged at 2200× *g* for 20 min. The collected serum was kept at −20 °C. By using a quantitative Chemiluminescence Enzyme-Linked Immunosorbent Assay, the Ultra-Sensitive serum estradiol (E2), progesterone (P4), and testosterone were quantitatively measured according to protocol (BQ Kits, Inc., San Diego, CA, USA, Catalogue Number BQ 561S). Sensitivity limits of E2 detection were 2.6 pg/mL and 0.23 ng/mL for P4 and 1.4 ng/mL.

### 2.10. Statistical Analysis

Data was fed to the computer and analyzed using the IBM SPSS software package version 20.0. (Armonk, NY, USA: IBM Corp.). The significance of the results obtained was judged at the 5% level. The data was presented as Mean + Standard Deviation (SD). A one-way ANOVA test was used to analyze the statistical differences within groups. Normally distributed quantitative variables (F-test, ANOVA) were applied, and the post hoc test (Tukey) was applied for pairwise comparisons. To determine if a sample of data comes from a normal distribution, the Shapiro–Wilk test is used. It assesses the normality of a dataset by comparing the observed data to the expected values of a normal distribution. The test calculates a W statistic that ranges from 0 to 1, with values closer to 1 indicating a better fit to a normal distribution. The null hypothesis is that the data is normally distributed, and the test determines if there is enough evidence to reject this hypothesis. In this test, a high *p*-value indicates the dataset has a normal distribution, while a low *p*-value indicates that it does not have a normal distribution. Homogeneity of variance is needed so that we can average the variances from each sample to estimate the population variance. For the homogeneity of variance, Levene’s test uses an F-test to test the null hypothesis that the variance is equal across groups. A *p*-value less than 0.05 indicates a violation of the assumption.

## 3. Results

### 3.1. Characterization Panel of Graphene Oxide

**Figure 1:**
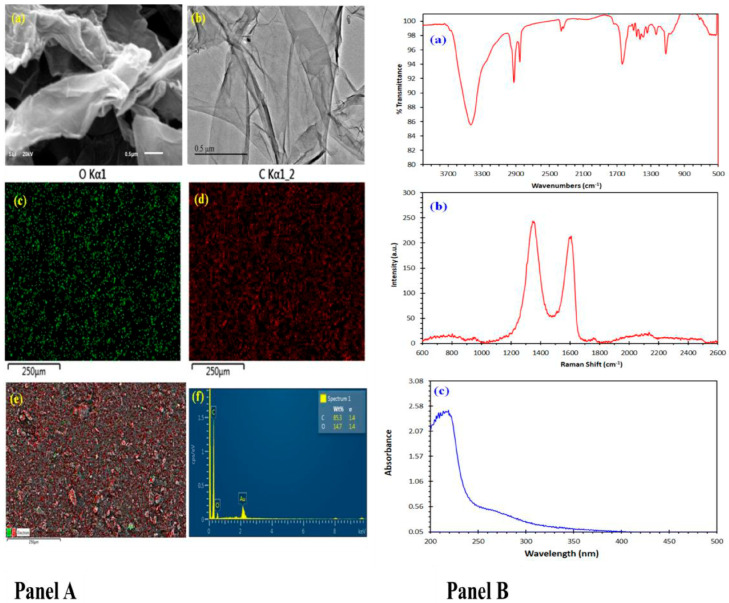
(**Panel A**) Morphological characterization of the prepared graphene oxide: (**a**) SEM image, (**b**) TEM image, (**c**) mapping image of oxygen, (**d**) mapping image of carbon, (**e**) combined mapping image of carbon and oxygen of graphene oxide, (**f**) EDX analysis of graphene oxide. (**Panel B**) Structural characterization of the prepared graphene oxide: (**a**) FT-IR spectrum of graphene oxide; (**b**) Raman spectrum of graphene oxide; (**c**) UV-Vis absorption spectrum of graphene oxide.

The morphology of graphene oxide was characterized using SEM and TEM. The SEM image (Figure 1a (Panel A)) indicates the formation of layers of graphene, and the layers are very thin, as can be seen from the TEM image in Figure 1b (Panel A). Figure 1c,d (Panel A) the mapping images of oxygen and the mapping image of carbon, respectively, while the mapping images of Figure 1e (Panel A) indicate the uniform distribution of oxygen functional groups on the graphene. The EDX spectrum (Figure 1f (Panel A)) indicates the elements forming the graphene, mainly carbon and oxygen, without the presence of any impurities.

Figure 1a (Panel B) reveals the FTIR spectrum of graphene oxide. In the FTIR spectrum of graphene oxide, two prominent bands appear at 2850 and 2920 cm^−1^, attributed to the symmetric and asymmetric stretching of the -CH_2_- groups. A characteristic stretching vibration peak of the hydroxyl group appears at 3430 cm^−1^. The sharp peak at 1608 cm^−1^ can be attributed to the C=C stretching vibration [40,41]. The FTIR spectra of graphene oxide also reveal bands at 2912 and 2840 cm^−1,^ which can be attributed to the C-H stretching vibrations of the -CH_2_.

The Raman spectrum of the prepared graphene oxide shows the main characteristic bands at 1350 and 1580 cm^−1^ (Figure 1b (Panel B)). The D-mode at 1350 cm^−1^ is attributed to the disordered structure of graphene [42]. The presence of disorder in sp2-hybridized carbon systems results in a resonance Raman spectrum and thus makes Raman spectroscopy one of the most sensitive techniques to characterize disorder in sp2 carbon materials. The G-mode is at about 1583 cm^−1^ due to the E2g mode. A G-band arises from the stretching of the C-C bond in graphitic materials and is common in all sp2 carbon systems. If there are some randomly distributed impurities or surface charges in the graphene, the G-peak can split into two peaks, a G-peak (1580 cm^−1^) and D’-peak (1620 cm^−1^). The reason could be that the localized vibrational modes of the impurities can interact with the extended phonon modes of graphene, resulting in the observed splitting. In our prepared graphene, there is no splitting, meaning no impurities. The chemical structure of graphene consists of benzene rings containing electrons, which display a UV–vis absorption peak at 230 nm (Figure 1c (Panel B)).

### 3.2. Maternal Toxicity

**Figure 2:**
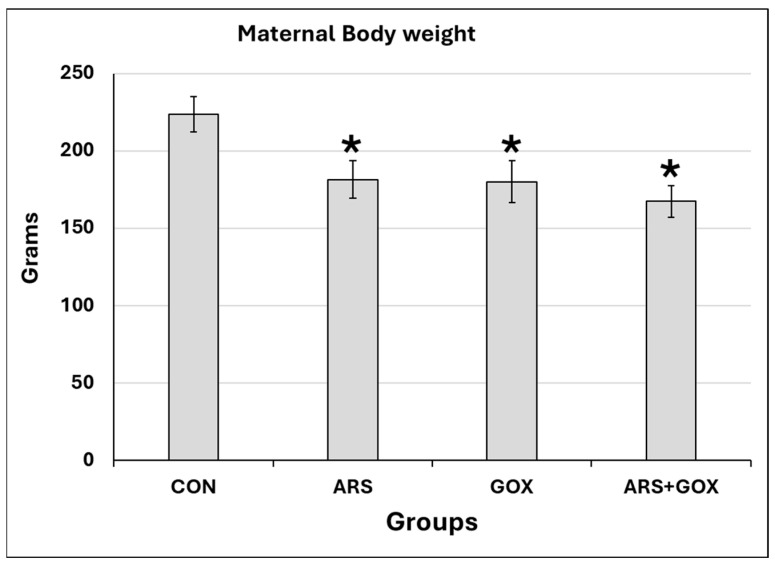
Maternal body weight after arsenic and/or graphene oxide exposure during the GD1 to GD20 period and the lactation period. Data expressed as mean ± SDM. (*n* = 15 female/group). * Significant difference as compared with the control group (*p* ≤ 0.05).

No obvious signs of unusual behaviour were recorded during GD1 to GD20, or the lactation period, compared with the control group. None of the pregnant female rats aborted between GD1 and GD20. No female rats were excluded from the study protocol. No differences in pregnancy duration or number of delivered offspring between the control and ARS-exposed groups were recorded. However, at weaning, a significant decrease in body weight was observed among experimental groups compared with the control group (Figure 2).

### 3.3. Offspring Toxicity from Birth to Puberty (PND 1–PND 90)

#### 3.3.1. Body Weight, Anogenital Distance, and Number of Nipples/Areolae

**Figure 3:**
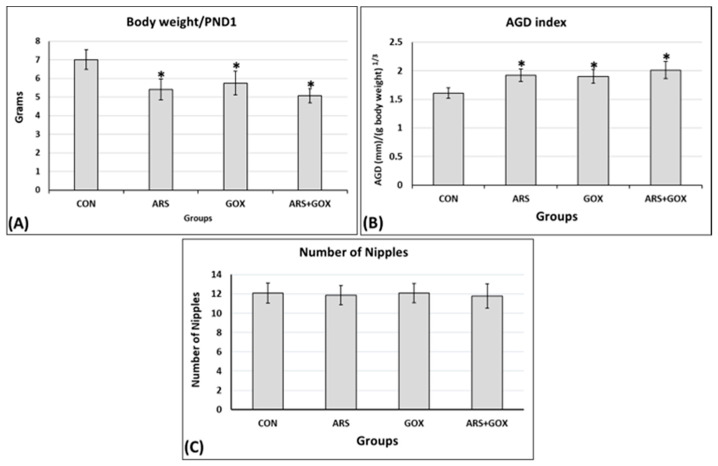
Parameters of female offspring at PND 1 exposed in utero and lactation to sodium arsenite and/or graphene oxide. (**A**) Body weight; (**B**) the relative anogenital distance; (**C**) the number of nipples (teats). Data expressed as mean ± SDM. (*n* = 30 offspring/group). * Significant difference as compared with the control group (*p* ≤ 0.05).

Perinatal and lactation treatment with arsenite and/or graphene oxide at PND 1 showed a significant reduction in body weight compared to the controls (*p* ≤ 0.05; Figure 3A). The relative anogenital distance (AGD) at PND 1 significantly increased in the female offspring exposed in utero and lactation to ARS, GOX, or ARS + GOX compared with the control group (*p* ≤ 0.05; Figure 3B). On PND 15, no significant changes were estimated between the arsenite- and/or graphene oxide-exposed groups and the control group in the number of teats (nipples of the mammary glands) or areolae (*p* ≤ 0.05; Figure 3C).

#### 3.3.2. Signs of Puberty Onset (Vaginal Opening and First Estrus)

**Figure 4:**
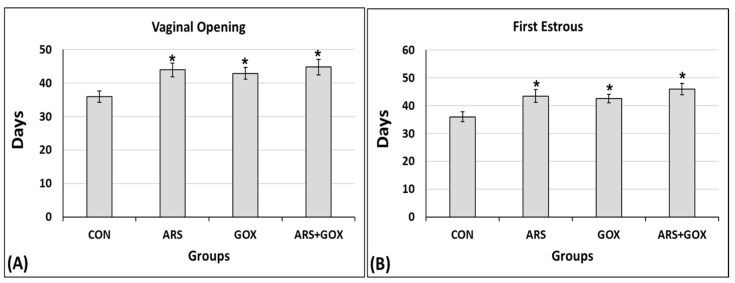
Vaginal opening and days of the first estrus of female offspring exposed to sodium arsenite and/or graphene oxide or water during the gestational and lactation period. (**A**) Day of the vaginal opening; (**B**) day of the first estrus. Data represent mean ± SDM (*n* = 30 offspring/group). * Significant difference as compared with the control group (*p* ≤ 0.05).

For puberty onset, the delay in the day of the vaginal opening and first estrus was significantly higher in the ARS, GOX, and co-exposed group (ARS + GOX) compared to controls (*p* ≤ 0.05; Figure 4A,B). On the first day of vaginal opening, PND 44, PND 43, and PND 45 were in the arsenite, graphene, and co-exposed arsenite + graphene groups, respectively, compared to PND 36 in the control group. In the arsenite + graphene co-exposure group, the graphene significantly aggravated the delay on the first day of estrous compared with female offspring in the arsenite and graphene alone, and control group (*p* ≤ 0.05; Figure 4B). In addition, all female offspring rats in the control group had complete vaginal canalization of the vagina on PND 42, but only 42%, 43%, and 41% of female offspring rats showed a synchronized vaginal canalization opening in the arsenite, graphene, and co-exposed arsenite + graphene group, respectively, at the same age. These findings demonstrated that the combination of arsenite with graphene oxide influenced both vaginal opening and the onset of the estrous cycle. Furthermore, 18% of the female offspring delivered in the combined arsenite and graphene oxide group exhibited imperfect vaginal openings at PND 85, while this figure was 22% and 20% in the arsenite and graphene groups, respectively.

#### 3.3.3. Estrous Cycle

At PND 90, the estrous cycle length was significantly reduced to less than 4–5 days, while the number of cycles showed a significant increase in the exposed groups compared to the control group (*p* ≤ 0.05; Table 1). The duration of the metestrus period per cycle was significantly decreased, while the duration of the diestrus period per cycle increased (*p* ≤ 0.05; Table 1).

#### 3.3.4. Sexual Behaviour and Receptivity of Female Offspring

**Figure 5:**
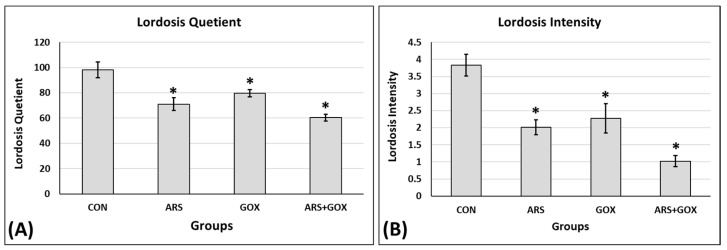
Assessment of copulatory behaviour (**A**) lordosis quotient and (**B**) ordosis intensity of the sexual receptivity/proestrus phase in female rats. Values expressed as mean ± SDM. (*n* = 25 offspring/group). * Significant difference as compared with the control group (*p* ≤ 0.05).

The ARS, GOX, and ARS + GOX cycling female rat offspring at proestrus presented significant decreases in mean lordosis frequency to 28%, 19%, and 39%, respectively, compared with the 98% frequency of lordosis of the control female offspring. Similarly, the lordosis intensity of the females that responded to the mounting of experienced stud males was decreased to 48%, 41%, and 73% for ARS, GOX, and ARS + GOX cycling female offspring, respectively, compared with the 4.25% intensity of lordosis of the control cycling female offspring (Figure 5). The combined ARS and GOX group showed an increase in the percentage of inhibition of reflex magnitude of lordosis, reflex magnitude 1, and reflex magnitude 2 for ARS and GOX alone, compared to a higher percentage of reflex magnitude 4 for the control (Figure 5).

#### 3.3.5. Fertility and Reproductive Performance

The gestation rate was significantly reduced in all exposed female rat offspring compared with the control female offspring (*p* ≤ 0.05; Table 2). Significant decreases were estimated in the fertility potential in all groups compared with the control group (*p* ≤ 0.05; Table 2). The number of implantation losses (pre-implantation and post-implantation) and the number of resorptions were significantly increased in all exposed female rat offspring compared with the control female offspring (*p* ≤ 0.05; Table 2). Moreover, a significant reduction in the number of viable fetuses was detected in the exposed groups compared with the control group (*p* ≤ 0.05; Table 2).

#### 3.3.6. Biometric Parameters of Female Offspring at PND 90

**Figure 6:**
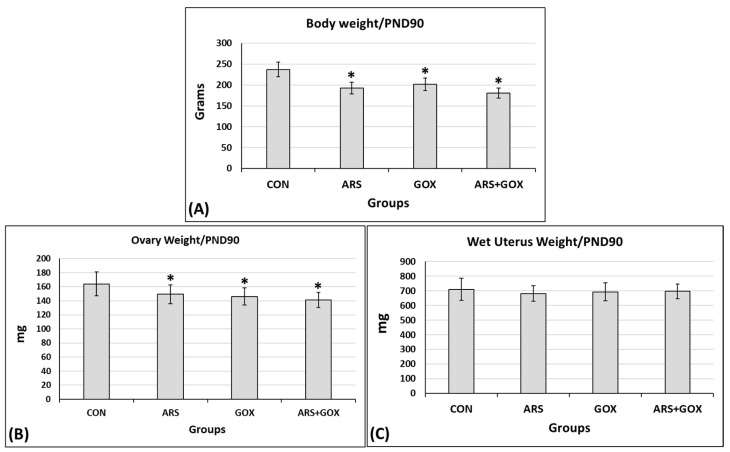
Biometric parameters (**A**) body weight, (**B**) ovary weight, and (**C**) wet uterus weight of female offspring rats at postnatal day 90 (PND 90) exposed to sodium arsenite and/or graphene oxide in comparison to the control group. Values expressed as mean ± SDM (*n* = 10 animals/group). * Significant difference as compared with the control group (*p ≤* 0.05).

At PND 90, body weight and wet ovarian weight in the exposed subgroups were significantly decreased compared to the control group. However, no significant differences were observed in uterine weight between the control and exposed groups (*p* ≤ 0.05, Figure 6).

#### 3.3.7. Status of Hormone Levels

On postnatal day 90, a significant decrease was observed in serum levels of estradiol.

**Figure 7:**
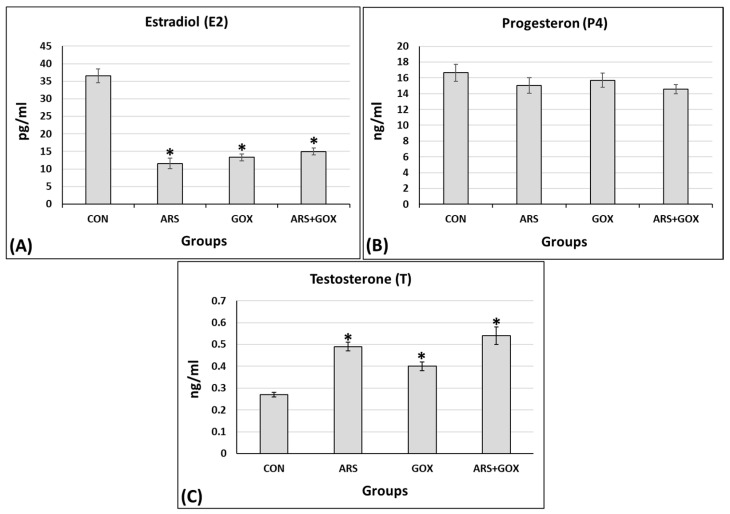
Serum estradiol (pg/mL), progesterone (ng/mL), and testosterone (ng/mL) levels from the female offspring exposed to in utero and lactation sodium arsenite and/or nano-graphene oxide compared with the control group on postnatal day 90. (**A**) Estradiol; (**B**) progesterone; (**C**) testosterone. Mean ± SEM. *n* = 10 offspring/group). * Significant difference as compared with the control group (*p* ≤ 0.05).

However, no significant changes were observed in serum levels of progesterone in the exposed groups compared with the control group (*p* ≤ 0.05, Figure 7A,B). Conversely, testosterone was significantly increased (*p* ≤ 0.05, Figure 7C).

## 4. Discussion

Notably, the rise in global exposure to environmental toxicants validates the occurrence of co-exposure to ARS-GOX, posing significant threats to public health. The developing crucial organs—and, subsequently, full growth and development—are extremely sensitive to xenobiotics or toxicants, particularly in utero and during lactation. This study simulates real environmental conditions to investigate the adverse effects in utero and during lactation exposure to ARS and/or GOX on the reproductive axis of female rat offspring.

During pregnancy and lactation, maternal exposure to ARS and/or GOX through drinking water induced maternal toxicity, as evidenced by decreased maternal body weight, which in turn led to prepubertal defects in offspring, without adverse effects on behaviour, pregnancy, or parturition. ARS and GOX individually disrupted pubertal parameters, including AGD, vaginal opening, first estrus, reproductive hormones, and cyclicity. When combined, these environmental toxicants exacerbated reproductive disorders through complementary mechanisms. The results highlight the significance of the environmental risks associated with the co-exposure of females to toxics and nanomaterials.

Numerous reports have suggested that nanoparticles enhance the toxicity of toxicants by boosting their uptake and bioavailability [43,44]. Other studies confirm that GOX can bind to toxicants, acting as a carrier to enhance their transport [45]. Moreover, the exacerbation and aggravation of GOX toxicity are attributed to the accumulation and deposition of toxicants in target tissues or organs due to its high amphiphilic affinity, which enables it to easily cross membrane barriers and enhance biomolecular permeability [46].

Despite no changes in the number of nipples, ARS and/or GOX alone increased AGD, referring to masculinization in female offspring [47]. This sexual retardation may be attributed to hormonal disruption, deterioration in the hypothalamic-pituitary-ovarian axis, or damage to one or more of these systems. Such issues may originate in the early stages of development.

Delayed puberty onset was frequently observed in the ARS and/or GOX exposure groups, while the most significant effects were evident in the co-exposure group. This delay may be associated with placental dysfunction and trophoblast cell damage, suggesting that the synergy of ARS and GOX may exacerbate impaired fetal development by altering maternal metabolism and, consequently, remodelling or reprogramming fetal development trajectories [48,49]. Moreover, the adhesion of GOX to the embryonic chorion through interactions of the hydroxyl group may indicate a delay in organ differentiation [50].

The combined mechanism of high binding affinity to the estrogen receptor α (ERα) [51] and mimicking of the effects of natural estradiol (E2) [52] may explain the decreased estradiol, which is crucial for puberty, causing delayed juvenile puberty or increasing sexual retardation [53]. Indeed, we can hypothesize that several pathways mediated the suppression of steroidogenic proteins/genes, such as AKT, PKA-ERK, CREB, JNK-cJUN, and MAPK, through multiple signalling cascades [54], playing a crucial role in reducing estradiol [55] and suggesting that these molecular signalling cascades of steroidogenesis are the most vulnerable to toxins [56].

Furthermore, in utero, the direct estrogen-interfering and endocrine-disrupting mechanism may increase the expression of genes responsible for regulating estrogen and estrogen receptors, which are involved in suppressing the pre- and post-pubertal development of the mammary gland [57] and delaying vaginal opening [58]. On the other hand, delayed or absent puberty may be assumed due to the extreme adverse effects of ARS on the loss of signalling function of kisspeptin, which is involved in the timing of puberty onset [59,60]. Interestingly, this suggests that kisspeptin is a target of ARS and GOX during puberty. Again, an impaired hypothalamus–pituitary–ovary axis and maternal homeostasis may result in the dysfunction and subsequent pathophysiology of offspring [61,62].

According to the detection of GOX in gametes, transferring nanoparticles to the offspring could explain the damage to the genitals of the offspring [63,64]. Taken together, the present findings indicate that the potential reproductive toxicity of genital retardation in the offspring was due to maternal exposure to GOX at environmentally relevant concentrations.

Further possible molecular mechanisms behind the reduction in ovarian steroids may be related to lowering CYP17A1 CYP17A1mRNA expression in theca-interstitial cells [65] and/or abrogating impairment in the intricate cooperative interactions between granulosa and theca cells [66], which are essential in the conversion of androgen to estrogen in granulosa cells and differentiation of theca cells via the regulation of gonadotropins [67]. CYP11A1, STAR, HSD17B1, CYP19A1, CYP17A1, and HSD3B1 are genes encoding for proteins implicated in steroid biosynthesis and ovarian development, so exposure to ARS and GOX, combined or alone, may be correlated with their downregulation [68]. Recently, Li et al. [69] reported that exposure to GOX resulted in damage to ovarian granulosa cells.

The succession of the estrous cycle was significantly disrupted, as evidenced by the alterations in the frequency of the metestrus and diestrus stages, the increase in the number of cycles, and the decrease in cycle length observed in the present findings.

Indeed, cycle disruption and irregularity are indicative of hypothalamus–pituitary–gonad disorders, a downregulation of steroidogenic genes, a degeneration of ovarian cellularity, and delayed follicular maturation induced by arsenic toxicity [70]. The disruption of cycle progression may also be due to the downregulation of the encoding patterns of Dhcr7, Akr1b7, and Kcne2 as robust biomarkers in the ovarian cortex during in utero gonadal development [71]. Additionally, retarded puberty parameters may be correlated with abnormal cyclicity and disrupted female steroidogenesis, both of which are essential for the growth and differentiation of reproductive organs [72].

The toxicity of ARS is exacerbated or aggravated in co-exposure with GOX, significantly more than with ARS or GOX alone, suggesting that GOX could boost ARS toxicity via synergistic effects. The triggered toxicity of ARS may also be related to the oxygenic functionality of the surface area and endocytosis-mediated cellular internalization of GOX [73]. The physicochemical properties and bioavailability characteristics of GOX may enhance its cellular internalization and, consequently, its interactions at the cellular level [74]. Furthermore, ARS being granted GOX-mediated adsorption and oxidation ability could potentially increase the accumulation of ARS, giving it various toxicity effects [75]. Due to the colloidal suspension-dependent surface area, the bioavailability of GOX is altered, influencing the toxicity of arsenic and resulting in changes in the reproductive physiology of rats [76].

Compared to the control group, offspring in the co-exposure group showed a significant reduction in pregnancy rates and the number of live births. Nanoparticles caused a decrease in fertilization rates and live birth numbers, and an increase in implantation loss [77]. Gestational exposure to GOX nanoparticles significantly decreases reproductive capabilities, suggesting that GOX can cause multigenerational harmful effects [78,79].

Understanding the environmental fate and transport of GO is complicated by its possible physical and chemical variations. In water, GOX tends to develop wrinkles and ripples to minimize the total free energy [80], and the designed GOX morphologies turn out to be a promising way to augment its properties. Further, the chemical transformation of GOX has been proven to be common in natural environments, such as photochemical reactions in sunlight [81] and reduction by bacteria [82]. Moreover, graphene oxide is readily dispersible in water because of hydrophilic oxygen groups located on the basal plane and the edges. A literature review revealed that GOX sheets can be dispersed due to their amphiphilic characteristics [83].

Therefore, the current findings suggest that the exacerbated effects of GOX can be attributed to the crucial interplay between its physical properties (crumpling and ζ-potential) and its chemical characteristics (carboxyl functional groups). Together, these factors significantly enhance the aqueous stability of GOX, whereas reductions in GOX have been shown to lead to much faster aggregation kinetics at lower ionic strengths.

## 5. Conclusions

The present findings systematically unravel the toxic effects of combined ARS and GOX on prepuberty, puberty, and maturity parameters. The perturbations observed in the current study were more significant in the combined-ARS-and-GOX offspring after maternal exposure during the gestation and lactation periods compared with those in offspring exposed to ARS or GOX alone.

Although the natural interactions between ARS and GOX may be more complex, the present findings reveal the possible role of GOX in exacerbating female prepuberty in combination with ARS in perinatal and lactation periods.

The observed adverse effects of arsenic and graphene oxide, whether alone or in combination, should be regarded as indicative of potential reproductive risks in humans, particularly under conditions of chronic or elevated exposure. However, species-specific differences in toxicokinetics, metabolism, and susceptibility may influence the degree of risk. Thus, our results should be interpreted as supportive experimental evidence rather than direct predictors of human outcomes.

We believe that our findings highlight a pressing need for further studies, including mechanistic research in human-relevant models and well-designed epidemiological investigations, to better establish human health risk assessment and safe exposure thresholds.

### Limitations of the Study

Although the present study provides important insights into the reproductive toxicity of arsenic and graphene oxide in female rats and their offspring, we expand on several limitations in this Limitations of the Study section to clarify the key constraints. Specifically, we acknowledge the following:

1. High Dose Levels: The doses of arsenic and graphene oxide administered in this study were relatively high compared to typical environmental or occupational exposure levels. While this approach is valuable for identifying potential toxic effects and mechanisms, the findings may not directly reflect the risks associated with lower, chronic, real-life exposures.
2. Single-Species Model: The use of female rats as an experimental model provides important information, but extrapolation to humans should be carried out cautiously. Differences in physiology, metabolism, and reproductive biology between rodents and humans may limit the generalizability of the results.
3. Exposure Duration and Route: The exposure duration and route (oral, intraperitoneal, etc.) may not fully mimic human exposure scenarios, where chronic low-dose exposure through water, food, or inhalation is more common. This difference may influence the distribution, bioaccumulation, and toxicokinetics of arsenic and graphene oxide.
4. Combined Exposure Assessment: While the study investigated co-exposure to arsenic and graphene oxide, the design may not capture the full complexity of interactive effects at lower, environmentally relevant concentrations. The observed toxicity could be amplified by the supraphysiological doses used.
5. Limited Mechanistic Exploration: Although the reproductive endpoints of offspring puberty and maturity were assessed, mechanistic insights at the molecular and cellular levels (e.g., oxidative stress pathways or epigenetic modifications) were not comprehensively investigated. Such analyses would be valuable for clarifying the underlying basis of the observed effects.
6. Long-Term Offspring Outcomes: The assessment of offspring was limited to early developmental and reproductive parameters. Long-term effects across the lifespan, including transgenerational impacts, were not evaluated and remain to be determined. In addition, the present study aimed to investigate the prepubertal, pubertal, and maturity endpoints of female offspring, which was conducted only in early developmental stages (short-term offspring outcomes).
7. Daily intake water consumption (mg/kg/day): Noting the absence of direct estimated intake data, we recommend that future studies incorporate the daily monitoring of water consumption to allow more accurate dose–response assessment.
8. Lack of Molecular and Histological Endpoints: A further limitation of the present study is the absence of direct molecular and histological assessments to support the proposed mechanistic pathways. While the reproductive and developmental outcomes observed suggest possible involvement of oxidative stress, endocrine disruption, and neuroendocrine signalling alterations associated with puberty and maturity, these mechanisms remain hypothetical without corroborating molecular markers or tissue-level evidence. Future studies incorporating gene expression profiling, protein analyses, and histopathological examinations of reproductive and neural tissues are warranted to validate and expand upon the mechanistic interpretations suggested here.
9. Analysis of Gonadotropins (LH, FSH): The inclusion of gonadotropins (LH and FSH) would have provided a more comprehensive assessment of hypothalamic–pituitary–gonadal (HPG) axis disruption. We also highlight that future work should include gonadotropin measurements alongside sex steroid hormones to better characterize the endocrine mechanisms underlying arsenic and graphene oxide toxicity.

## Figures and Tables

**Table 1:** Effects in utero, during the lactation period, and after drinking water exposure on the four phases of the estrous cycle (three consecutive cycles/15 days) in control and ARS, GOX, and ARS + GOX-treated rats.

	Experimental Groups			
	CON	ARS	GOX	ARS + GOX
(%) Females with regular estrous cycles	100	33	15	25
Number of days in estrous cycle	5.13± 0.83	4.63 ± 0.71 *	4.56 ± 0.29 *	4.53 ± 0.21 *
Number of estrous cycles	2.94 ± 0.11	3.54 ± 0.12 *	3.51 ± 0.32 *	3.61 ± 0.12 *
Proestrus/cycle	1.11 ± 0.013	1.00 ± 0.013	1.00± 0.033	1.09 ± 0.011
Estrus/cycle	1.25 ± 0.042	1.11 ± 0.012	1.09 ± 0.012	1.07 ± 0.21
Metestrus/cycle	1.30± 0.032	0.52 ± 0.02 *	0.45 ± 0.022 *	0.34 ± 0.10 *
Diestrus/cycle	1.47 ± 0.015	2.00 ± 0.75 *	2.02 ± 0.55 *	2.03 ± 0.21 *

Values expressed as mean ± SDM (*n* = 25 offspring/group). * Significant difference as compared with the control group (*p* ≤ 0.05). ANOVA followed by Tukey test.

**Table 2:** Fertility and reproductive performance of female offspring exposed to sodium arsenite and/or graphene oxide compared with controls.

Parameters	Experimental Groups			
	CON	ARS	GOX	ARS + GOX
% Gestation rate	100	79 *	77 *	58 *
Fertility potential (efficiency of implantation)	97.47 ± 4.36	74.12 ± 3.11 *	72.79 ± 3.88 *	66.37 ± 4.66 *
Corpora lutea number/uterus	13.43 ± 1.42	11.67 ± 2.32 *	11.27 ± 1.83 *	10.02 ± 1.56 *
Implantation number/uterus	13.09 ± 1.66	8.65 ± 1.57 *	9.33 ± 0.68 *	6.65 ± 0.48 *
Live litter size (viable number)	12.78 ± 2.64	6.10 ± 0.52 *	7.02 ± 0.68 *	4.05 ± 0.68 *
Stillborn number	0.0	1.10 ± 0.01 *	1.14 ± 0.05 *	1.13 ± 0.73 *
Resorption number	0.31 ± 0.01	1.45 ± 0.02 *	1.17 ± 0.02 *	1.47 ± 0.49 *
Pre-implantation loss (%)	2.53 ± 0.62	25.88 ± 0.60 *	17.214 ± 0.92 *	33.63 ± 2.61 *
Post-implantation loss (%)	2.37 ± 0.01	29.47 ± 2.02 *	24.76 ± 2.11 *	39.09 ± 1.32 *
Fetus weight (g)	3.78 ± 0.21	3.59 ± 0.21	3.63 ± 0.21	3.62 ± 0.21
Crown rump length (CRL) (mm)	41.01 ± 2.12	40.01 ± 2.45	38.02 ± 1.82	38.41 ± 1.23
Placenta weight (mg)	450 ± 70.13	430 ± 69.54	440 ± 71.34	400 ± 66.65

Values expressed as the mean ± SDM. *n* = 20 offspring/group. * Significant difference as compared with the control group (*p* ≤ 0.05).

## Data Availability

The data that support the findings of this study are not available from the corresponding author upon reasonable request.
